# Real-world outcomes of CDK4/6 inhibitors in combination with endocrine therapy as first-line treatment for HR-positive/HER2-negative advanced breast cancer: multicenter evidence from China

**DOI:** 10.1186/s12885-026-16176-y

**Published:** 2026-05-20

**Authors:** Xianan Guo, Panni Li, Shiyao Hu, Yuqin Ding, Bojian Xie, Yangjun Cai, Huihui Chen, Xiawei Li, Shiwei Guo, Yanyan Wang, Yunxiang Zhou, Yiding Chen

**Affiliations:** 1 Department of Breast Surgery and Oncology, The Second Affiliated Hospital, Zhejiang University School of Medicine, Hangzhou, Zhejiang China; 2 Cancer Institute (Key Laboratory of Cancer Prevention and Intervention, China National Ministry of Education), The Second Affiliated Hospital, Zhejiang University School of Medicine, Hangzhou, China; 3 Zhejiang Cancer Hospital, Hangzhou Institute of Medicine (HIM), Chinese Academy of Sciences, Hangzhou, Zhejiang China; 4 Department of Thyroid and Breast Surgery, Taizhou Hospital of Zhejiang Province Affiliated to Wenzhou Medical University, Taizhou, China; 5 Department of Epidemiology, Harvard T.H. Chan School of Public Health, Harvard University, Boston, MA USA; 6 Department of General Surgery, The Second Affiliated Hospital, Zhejiang University School of Medicine, Hangzhou, Zhejiang China; 7 Linhai First People’s Hospital, Taizhou, Zhejiang China

**Keywords:** Advanced breast cancer, HR-positive/HER2-negative, Real-world evidence, Cyclin-dependent kinase 4/6 inhibitor, Palbociclib, Abemaciclib, Dalpiciclib

## Abstract

**Background:**

CDK4/6 inhibitors combined with endocrine therapy constitute the global first-line standard of care for HR + /HER2 − advanced breast cancer (ABC), yet no head-to-head trials comparing different CDK4/6 inhibitors have been conducted, and real-world comparative data in Chinese patients is currently unavailable.

**Methods:**

We conducted a multicenter retrospective study of 341 Chinese patients with HR + /HER2 − ABC who received first-line palbociclib (*n* = 177), abemaciclib (*n* = 114), or dalpiciclib (*n* = 50) from 2018 to 2023. The primary endpoint was progression-free survival (PFS); secondary endpoints included safety, tolerability, and objective response rate.

**Results:**

Median PFS was 24 months for palbociclib and 32 months for abemaciclib, while median PFS for dalpiciclib was not reached. Abemaciclib significantly prolonged PFS compared with palbociclib (palbociclib vs. abemaciclib: adjusted HR = 1.86, 95% CI 1.24–2.79), with consistent benefits in premenopausal, postoperative recurrence, progesterone receptor–positive, HER2-low, luminal B, visceral metastasis, and endocrine-resistant subgroups (all *p* < 0.05). Overall tolerability was comparable, though palbociclib and dalpiciclib were associated with higher myelosuppression, whereas abemaciclib more frequently caused low-grade diarrhea.

**Conclusions:**

This is the larger real-world study to compare different CDK4/6 inhibitors in Chinese HR + /HER2 − ABC patients. Abemaciclib demonstrated a significant PFS advantage over palbociclib while maintaining a manageable safety profile, particularly benefiting biologically aggressive or treatment-resistant subgroups. These findings provide population-specific evidence to guide individualized CDK4/6 inhibitor selection and refine first-line treatment strategies for Chinese patients with HR + /HER2 − ABC.

**Supplementary Information:**

The online version contains supplementary material available at 10.1186/s12885-026-16176-y.

## Introduction

Hormone receptor-positive, human epidermal growth factor receptor 2-negative (HR +/HER2 −) subtype accounts for the highest proportion (~ 60%) in advanced breast cancer (ABC), yet the median overall survival (OS) remains only about 36 months [1]. Historically, the high resistance rates to conventional chemotherapy and endocrine therapy (ET) created a bottleneck in improving survival outcomes for these patients.

In recent years, large randomized controlled trial (RCT) series, including the PALOMA, MONALEESA, MONARCH, and DAWNA studies, have demonstrated that in patients with HR +/HER2 − ABC, combining CDK4/6 inhibitors (palbociclib, ribociclib, abemaciclib, or dalpiciclib) with ET significantly prolongs progression-free survival (PFS) compared with ET alone, with ribociclib and abemaciclib also showing OS benefits [2–5]. Accordingly, international and Chinese clinical guidelines now recommend CDK4/6 inhibitors in combination with aromatase inhibitors or fulvestrant as standard first-line therapy for HR +/HER2 − ABC [6, 7]. In China, palbociclib, abemaciclib, ribociclib, and dalpiciclib have all been approved. However, owing to the lack of head-to-head comparisons, current guidelines do not favor one CDK4/6 inhibitor over another.

Existing real-world studies comparing the efficacy and safety of different CDK4/6 inhibitors are predominantly based on Western populations [8–11]. Yet clinical trial subgroup analyses suggest potential racial differences. For instance, PALOMA-2 [12, 13], MONARCH-2 [14, 15], and MONARCH-3 [16] showed longer PFS in Asian patients compared with the overall cohort or White patients, while MONALEESA-7 [17] showed more pronounced OS benefit in Asian than in non-Asian patients. Such variations may reflect underlying differences in genetic profiles, epidemiology, or dietary habits, underscoring the importance of evaluating CDK4/6 inhibitors in specific racial groups. Importantly, the beneficiary profiles of these agents in the Chinese population remain unclear.

To address this knowledge gap, we conducted a multicenter retrospective study in China to compare the efficacy, safety, and tolerability of palbociclib, abemaciclib, and dalpiciclib (ribociclib was not included as it is less frequently used in China) as first-line therapy for HR +/HER2 − ABC, and to identify subgroups most likely to benefit. These findings may help refine treatment selection and provide real-world evidence to guide individualized clinical decision-making for Chinese patients with HR +/HER2 − ABC.

## Methods

### Study design and patient population

This multi-center retrospective study was an observational, non-interventional study involving patients with HR +/HER2 − ABC who received CDK4/6 inhibitors as first-line treatment between 2018 and 2023. Patients were followed until the end of 2024. This study was approved by the ethics committee of each participating hospital.

Eligible patients were required to be histologically diagnosed with HR +/HER2 − ABC (including unresectable locally advanced and metastatic breast cancer). Those who received at least one cycle of treatment with CDK4/6 inhibitors (palbociclib, abemaciclib or dalpiciclib) plus ET as first-line treatment and were followed up on for at least half a year were selected. First-line treatment was defined as the first systemic treatment regimen that patients received for advanced disease.

### Data source and outcomes

Demographic, clinicopathologic, and treatment patterns were extracted from electronic medical records. Toxicity data and patient outcome were collected through clinician documentation or telephone encounters.

### Outcomes

The primary outcome was PFS, and the secondary outcomes were safety (incidence of adverse events), tolerability (incidence of dose reduction or discontinuation due to adverse events) and objective response rate (ORR). The PFS was defined as the time from the start of CDK4/6 inhibitors to disease progression or death from any cause. The ORR was defined as the proportion of patients with complete response and partial response.

### Statistical analysis

Normally distributed continuous variables were presented as mean and standard deviation, with intergroup differences compared by ANOVA analysis; non-normally distributed continuous variables were presented as median and interquartile range, with intergroup differences analyzed through Kruskal–Wallis analysis; categorical variables were presented as percentages, with intergroup differences assessed by Pearson's chi-square test or Fisher's test, as appropriate.

Survival analyses were conducted using the Kaplan–Meier method and compared through log-rank test. Adjusted hazard ratio (aHR) and 95% confidence interval (CI) were calculated by multi-variable Cox proportional hazard model. Subgroup analyses were conducted based on key stratification factors. In addition, propensity score matching (PSM) was performed to balance baseline characteristics between palbociclib group and abemaciclib group (dalpicilcib group was not suitable due to limited sample size). The propensity score model included the following covariates: age, menopausal status, disease status, ER/PR expression, HER2 status, molecular subtype, bone-only metastasis, visceral metastasis and endocrine resistance.

Analyses were conducted using SPSS version 26.0, RStudio version 2022.07.0 and GraphPad Prism version 9.5.1.

## Results

### Patient characteristics

**Fig. 1:**
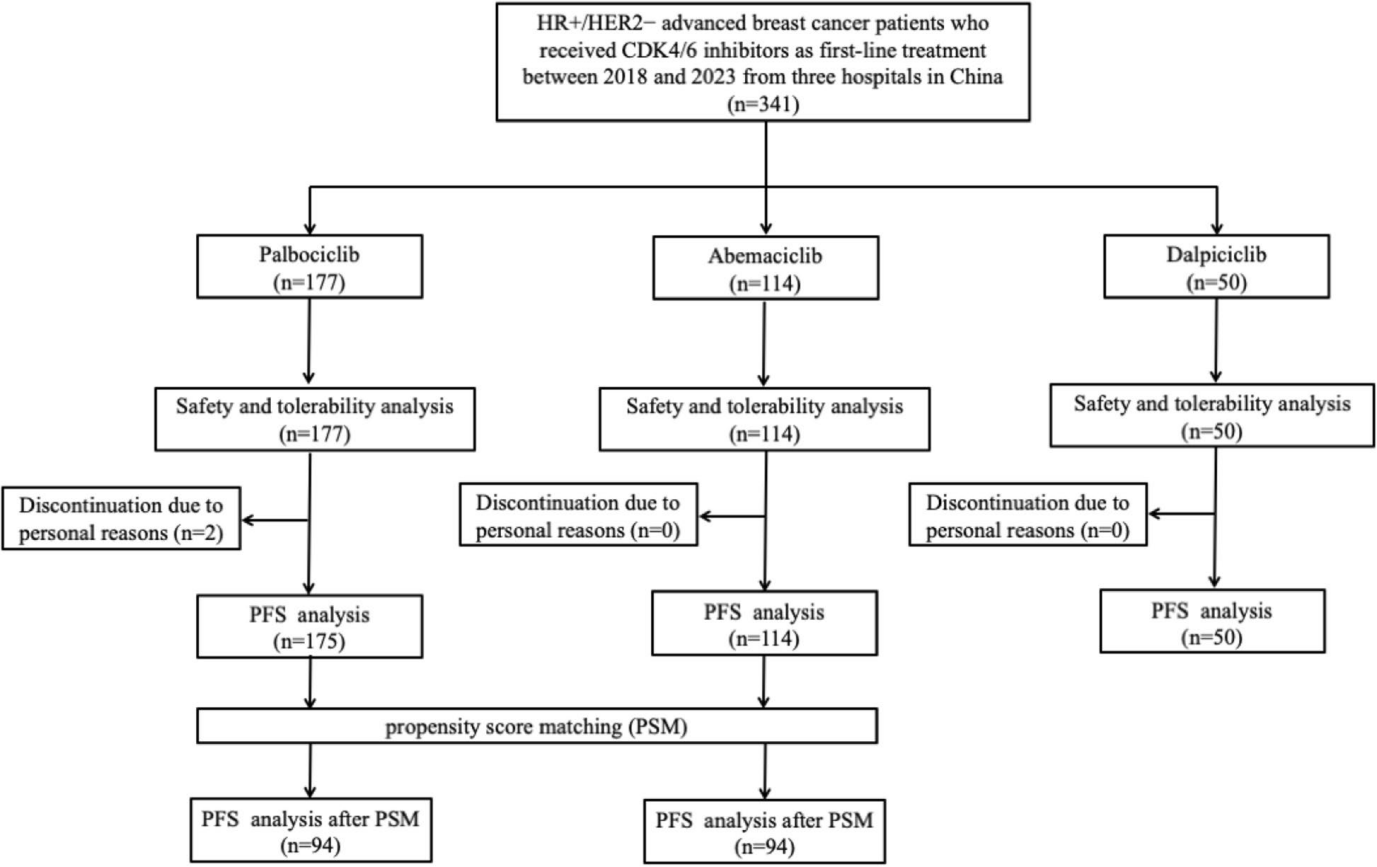
Patient attrition diagram

A total of 341 HR +/HER2 − ABC patients treated with CDK4/6 inhibitors was enrolled in the study between 2018 and 2023. The median follow-up time for all patients was 28 months. Among them, 177 patients received palbociclib, 114 received abemaciclib, and 50 received dalpiciclib, all of whom were included in the safety and tolerability analysis. Two patients were excluded in PFS analysis due to discontinuation caused by personal reasons. Patient attrition diagram is shown in Fig. 1.

The mean ages of patients in the ​​palbociclib​​, ​​abemaciclib​​, and ​​dalpiciclib​​ groups were ​​56.0 ± 11.9​​, ​​56.8 ± 11.7​​, and ​​57.9 ± 10.8​​ years, respectively (Table 1). Most enrolled patients were ​​postmenopausal​​, ​​postoperative recurrence ​​, and luminal B-type​​ breast cancer. The baseline characteristics were ​​generally balanced​​ among the three groups, with differences observed only in the proportion of ​​postoperative recurrence, the incidence of ​​liver metastases and the rate of ​​visceral metastases​​. Notably, the ​​dalpiciclib group​​ had a ​​higher proportion of postoperative recurrence​​ (96.0%) compared to the ​​palbociclib (80.8%)​​ and ​​abemaciclib (84.2%)​​ groups. Patients receiving ​​palbociclib​​ showed a ​​higher prevalence of liver metastases and visceral metastases​​ than those in the ​​abemaciclib​​ and ​​dalpiciclib​​ groups.

**Table 1:** Baseline characteristics of patients in different groups

Characteristic	Palbociclib	Abemaciclib	Dalpiciclib	*p* value
Number of patients (n)	177	114	50	
Age (y,$\overline{\mathrm{x}}\pm \text{s }$)	56.0 ± 11.9	56.8 ± 11.7	57.9 ± 10.8	0.570
BMI (kg/m^2^,$\overline{\mathrm{x}}\pm \text{s }$)	23.4 ± 3.3	23.1 ± 3.4	24.2 ± 2.8	0.183
Menopausal status (n, %)				
Premenopausal	49 (27.7)	25 (21.9)	12 (24.0)	0.710
Postmenopausal	126 (71.2)	88 (77.2)	37 (74.0)	
Male	2 (1.1)	1 (0.9)	1 (2.0)	
Disease status (n, %)				
Recurrence	143 (80.8)	96 (84.2)	48 (96.0)	0.034*
Stage IV at diagnosis	34 (19.2)	18 (15.8)	2 (4.0)	
Metastatic sites (n, %)				
Bone	102 (57.6)	71 (62.3)	26 (52.0)	0.451
Bone-only	31(17.5)	37 (32.5)	18 (36.0)	0.003**
Lung	62 (35.0)	31 (27.2)	15 (30.0)	0.360
Liver	35 (19.8)	15 (13.2)	2 (4.0)	0.018*
Brain	3 (1.7)	1 (0.9)	1 (2.0)	0.843
Viscera	92 (52.0)	45 (39.5)	18 (36.0)	0.039*
ER(%)(M, Q)^#^	90 (70, 90)	90 (80, 90)	90 (80, 90)	0.172
PR(%)(M, Q)^#^	30 (1, 30)	30 (0, 80)	58 (6, 80)	0.225
Ki-67(%)(M, Q)^#^	25 (10, 40)	20 (15, 40)	30 (19, 33)	0.798
HER2(n, %)^#^				
0	57 (32.2)	43 (37.7)	14 (28.0)	0.422
1 +/2 +	120 (67.8)	71 (62.3)	36 (72.0)	
Molecular subtype (n, %)^#^				
Luminal	177 (100.0)	114 (100.0)	50 (100.0)	/
Luminal A	40 (22.6)	21 (18.4)	9 (18.0)	0.615
Luminal B	118 (66.7)	79 (69.3)	37 (74.0)	0.603
Endocrine resistance (n, %)				
No	99 (55.9)	56 (49.1)	28 (56.0)	0.131
Yes	78 (44.1)	58 (50.9)	22 (44.0)	
Primary resistance	24 (13.6)	23 (20.2)	13 (26.0)	
Secondary resistance	54 (30.5)	35 (30.7)	9 (18.0)	
Endocrine backbone (n, %)				
Aromatase inhibitor	122 (68.9)	72 (63.2)	29 (58.0)	0.296
Fulvestrant	55 (31.1)	42 (36.8)	21 (42.0)	
Prior neoadjuvant/adjuvant chemotherapy (n, %)				
Yes	126 (71.2)	83 (72.8)	36 (72.0)	0.956
No	51 (28.8)	31 (27.2)	14 (28.0)	

# For ER, PR, Ki-67, and HER2 status and molecular subtype, histopathological data from metastatic sites were used preferentially over primary tumor data when available. All cases with HER2 IHC 2+ were negative for amplification via in situ hybridization

* *p* <0.05; ** *p* <0.01

### Progression-free survival

#### Overall cohort

**Fig. 2:**
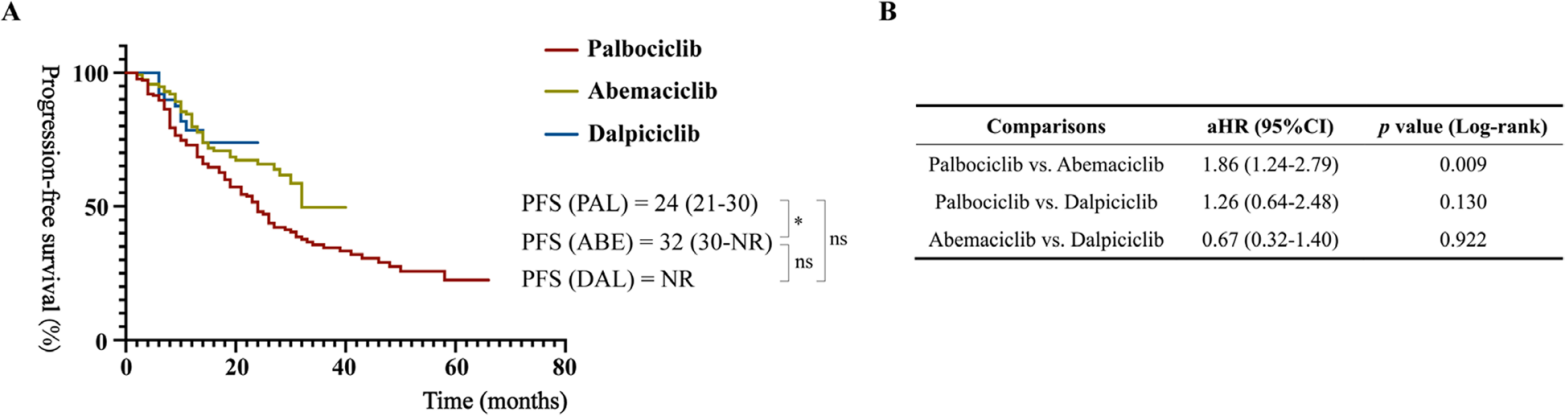
Progression-free survival (PFS) comparison of three CDK4/6 inhibitors as first-line therapy in advanced breast cancer.​​ **A**​​ Kaplan–Meier curves of PFS for the three CDK4/6 inhibitors. **B**​​ Adjusted hazard ratios (aHR) with 95% confidence intervals (CI) for the pairwise comparisons of PFS between the three CDK4/6 inhibitors

In the whole cohort, the median PFS was ​​24 months (95% CI, 21–30)​​ in the palbociclib group and ​​32 months (95% CI, 30-not reached [NR])​​ in the abemaciclib group, while the median PFS had ​​not yet been reached​​ in the dalpiciclib group (Fig. 2A). A ​​statistically significant difference​​ in PFS was observed between the palbociclib and abemaciclib groups (​​aHR = 1.86; 95%CI, 1.24–2.79; *p* = 0.009​​), whereas no significant differences were found in other comparisons (​​Fig. 2B​​). Numerically​​, patients treated with ​​abemaciclib​​ demonstrated the ​​greatest PFS benefit​​, followed by ​​dalpiciclib.

**Fig. 3:**
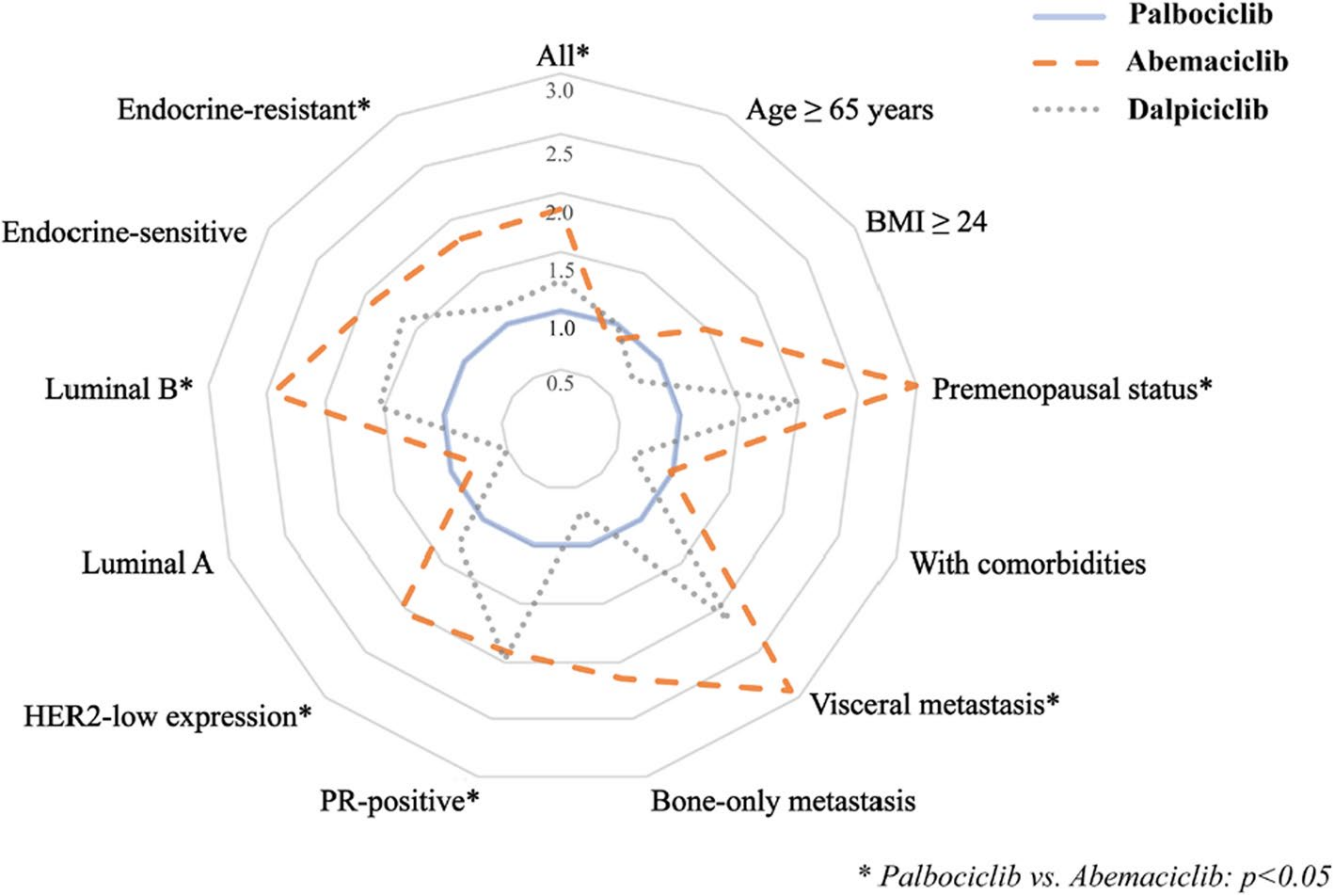
Radar chart of subgroup analyses for progression-free survival (PFS) with three CDK4/6 inhibitors. The plot presents adjusted hazard ratios (aHR) for PFS comparing palbociclib versus abemaciclib and palbociclib versus dalpiciclib across various patient subgroups

To further characterize the patient subgroups that may benefit from different CDK4/6 inhibitors, we conducted subgroup analyses comparing PFS among the three agents. In patients with age < 65 years, BMI < 24, premenopausal status, postoperative recurrence, progesterone receptor (PR) positivity, HER2-low expression, luminal B subtype, visceral metastases, or endocrine-resistant disease, abemaciclib significantly prolonged PFS compared with palbociclib (*p* < 0.05). However, in these same subgroups, no statistically significant differences in PFS were observed between dalpiciclib and the other two agents, although numerical trends suggested that dalpiciclib might provide slightly better PFS benefit than palbociclib (Fig. 3; supplementary Figs. 1 and 2).

In subgroups with age ≥ 65 years, BMI ≥ 24, postmenopausal status, comorbidities, PR negativity, HER2-0 expression, luminal A subtype, bone-only metastases, or endocrine-sensitive disease, no statistically significant differences in PFS prolongation were observed among the three agents. Numerically, abemaciclib and dalpiciclib showed marginally better PFS benefits than palbociclib in subgroups with age ≥ 65 years, postmenopausal status, HER2-0 expression, or endocrine-sensitive disease. Conversely, in subgroups with BMI ≥ 24, comorbidities, PR-negative status, luminal A subtype, or bone-only metastases, abemaciclib and palbociclib demonstrated slightly superior PFS benefits compared to dalpiciclib.

### Cohort after PSM

To balance baseline characteristics between palbociclib group and abemaciclib group, PSM was performed in a 1:1 ratio between the two groups (the dalpiciclib group was excluded due to insufficient sample size). A total of 94 patients were included in each group after matching, and no significant differences in baseline clinicopathological characteristics were observed between the two groups (Table 2).

**Table 2:** Baseline characteristics of patients in different groups after propensity score matching

Characteristic	Palbociclib	Abemaciclib	*p* value
Number of patients (n)	94	94	
Age (n, %)			
< 65 years	66	68	0.747
≥ 65 years	28	26	
BMI (kg/m^2^) (n, %)			
< 24	62	65	0.640
≥ 24	32	29	
Menopausal status (n, %)			
Premenopausal	22	24	0.579
Postmenopausal	71	70	
Male	1	0	
Disease status (n, %)			
Stage IV at diagnosis	14	16	0.488
DFS < 24months	11	16	
DFS ≥ 24months	69	62	
Metastatic sites (n, %)			
Lung	29	27	0.750
Liver	15	13	0.682
Bone-only	17	22	0.368
Viscera	44	38	0.378
ER (n, %)			
< 50%	3	7	0.194
≥ 50%	91	87	
PR (n, %)			
negative	23	25	0.738
positive	71	69	
HER2(n, %)			
0	29	33	0.535
1 +/2 +	65	61	
Molecular subtype (n, %)			
Luminal A	20	18	0.684
Luminal B	66	69	
Endocrine resistance (n, %)			
No	44	48	0.559
Yes	50	46	
Endocrine backbone (n, %)			
Aromatase inhibitor	60	61	0.879
Fulvestrant	34	33	

**Fig. 4:**
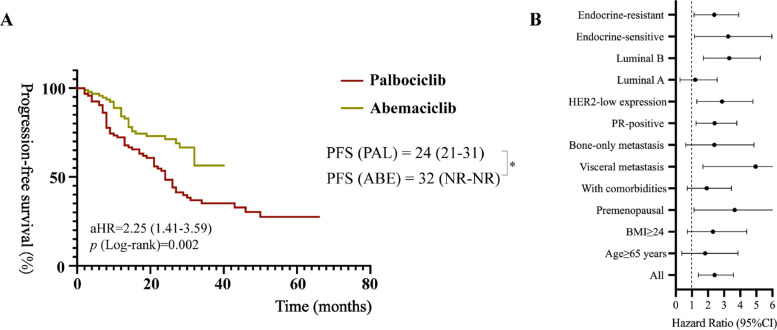
Comparison of progression-free survival (PFS) between two CDK4/6 inhibitors as first-line therapy for advanced breast cancer after propensity score matching.**​​ A**​​ Kaplan–Meier curves of PFS for the two CDK4/6 inhibitors after propensity score matching. **B**​​ Forest plot of adjusted hazard ratios (aHR) for PFS across various patient subgroups after propensity score matching

In the PSM-matched cohort, the abemaciclib group demonstrated significantly longer PFS compared to the palbociclib group (palbociclib vs. abemaciclib: aHR = 2.25, 95% CI 1.41–3.59) (Fig. 4A), which was consistent with the results observed in the overall study cohort (Fig. 2). Additionally, subgroup analyses were also conducted in the PSM-matched cohort, reaffirming that abemaciclib outperformed palbociclib in the following subgroups: premenopausal patients, those with visceral metastases, PR-positive status, HER2-low expression, luminal B subtype, and endocrine-resistant disease (*p* < 0.05). In contrast, no significant difference in efficacy was observed between the two drugs in patients aged ≥ 65 years, those with bone-only metastases, or luminal A subtype (*p* > 0.05) (Fig. 4B). These findings further validated the results obtained from the overall cohort (Fig. 3).

To explore factors influencing the efficacy of CDK4/6 inhibitors as first-line therapy in HR +/HER2 − ABC patients, we conducted univariate Cox regression analysis. The results indicated that the following factors potentially affected PFS (*p* < 0.05): type of CDK4/6 inhibitor, age, disease status, ER expression status, PR expression status, presence of liver metastases, endocrine sensitivity status, and type of concomitant ET. These variables were subsequently included in multivariate analysis, which revealed that the type of CDK4/6 inhibitor and presence of liver metastases were independent predictors of PFS (Supplementary Table 1). Abemaciclib was associated with better PFS, whereas liver metastases were correlated with poorer PFS.

### Objective response rate (ORR)

According to RECIST version 1.1 criteria, measurable lesions existed in 168 patients in the palbociclib group, 102 in the abemaciclib group, and 47 in the dalpiciclib group. The ORR for first-line advanced therapy were 45.8%, 41.2%, and 40.4%, respectively (*p* = 0.674) (Supplementary Fig. 3).

### Safety and tolerability

Among patients receiving CDK4/6 inhibitors in the first-line setting, the most frequent adverse event in the palbociclib group was neutropenia (83.6%), which also represented the most common grade 3/4 adverse event (44.6%) (Table 3). In the abemaciclib group, diarrhea occurred most frequently (76.3%), followed by neutropenia (70.2%), though neutropenia remained the predominant grade 3/4 adverse event (21.9%). The dalpiciclib group exhibited a higher incidence of hematologic toxicities, with neutropenia (86.0%), anemia (56.0%), and thrombocytopenia (22.0%) reported. Comparative analysis revealed that both palbociclib and dalpiciclib groups had significantly higher rates of neutropenia than the abemaciclib group (palbociclib vs. abemaciclib: *p* = 0.007; dalpiciclib vs. abemaciclib: *p* = 0.031). Conversely, abemaciclib was associated with significantly elevated incidences of diarrhea and increased creatinine levels (76.3%; 34.2%) compared to palbociclib (9.0%; 22.0%) and dalpiciclib (8.0%; 16.0%) (*p* < 0.05).

**Table 3:** Adverse events for three CDK4/6 inhibitors as first-line therapy in advanced breast cancer

Adverse events (AE)	Palbociclib (*n* = 177)			Abemaciclib (*n* = 114)			Dalpiciclib (*n* = 50)		
	Any grade	Grade 1–2	Grade 3–4	Any grade	Grade 1–2	Grade 3–4	Any grade	Grade 1–2	Grade 3–4
Neutropenia (n, %)	148 (83.6)	69 (39.0)	79 (44.6)	80 (70.2)	55 (48.2)	25 (21.9)	43 (86.0)	13 (26.0)	30 (60.0)
Anaemia (n, %)	62 (35.0)	45 (25.4)	17 (9.6)	51 (44.7)	39 (34.2)	12 (10.5)	28 (56.0)	27 (54.0)	1 (2.0)
Thrombocytopenia (n, %)	33 (18.6)	24 (13.6)	9 (5.1)	27 (23.7)	23 (20.2)	4 (3.5)	11 (22.0)	10 (20.0)	1 (2.0)
Elevated transaminase (n, %)	48 (27.1)	48 (27.1)	0 (0)	34 (29.8)	34 (29.8)	0 (0)	12 (24.0)	12 (24.0)	0 (0)
Elevated creatinine(n, %)	39 (22.0)	39 (22.0)	0 (0)	39 (34.2)	39 (34.2)	0 (0)	8 (16.0)	8 (16.0)	0 (0)
Diarrhea (n, %)	16 (9.0)	16 (9.0)	0 (0)	87 (76.3)	79 (69.3)	8 (7.0)	4 (8.0)	4 (8.0)	0 (0)
Rash (n, %)	18 (10.2)	18 (10.2)	0 (0)	17 (14.9)	17 (14.9)	0 (0)	5 (10.0)	5 (10.0)	0 (0)
Dose reduction due to AE (n, %)	33 (18.6)			21 (18.4)			12 (24.0)		
Discontinuation due to AE (n, %)	4 (2.3)			13 (11.4)			0 (0)		

**Fig. 5:**
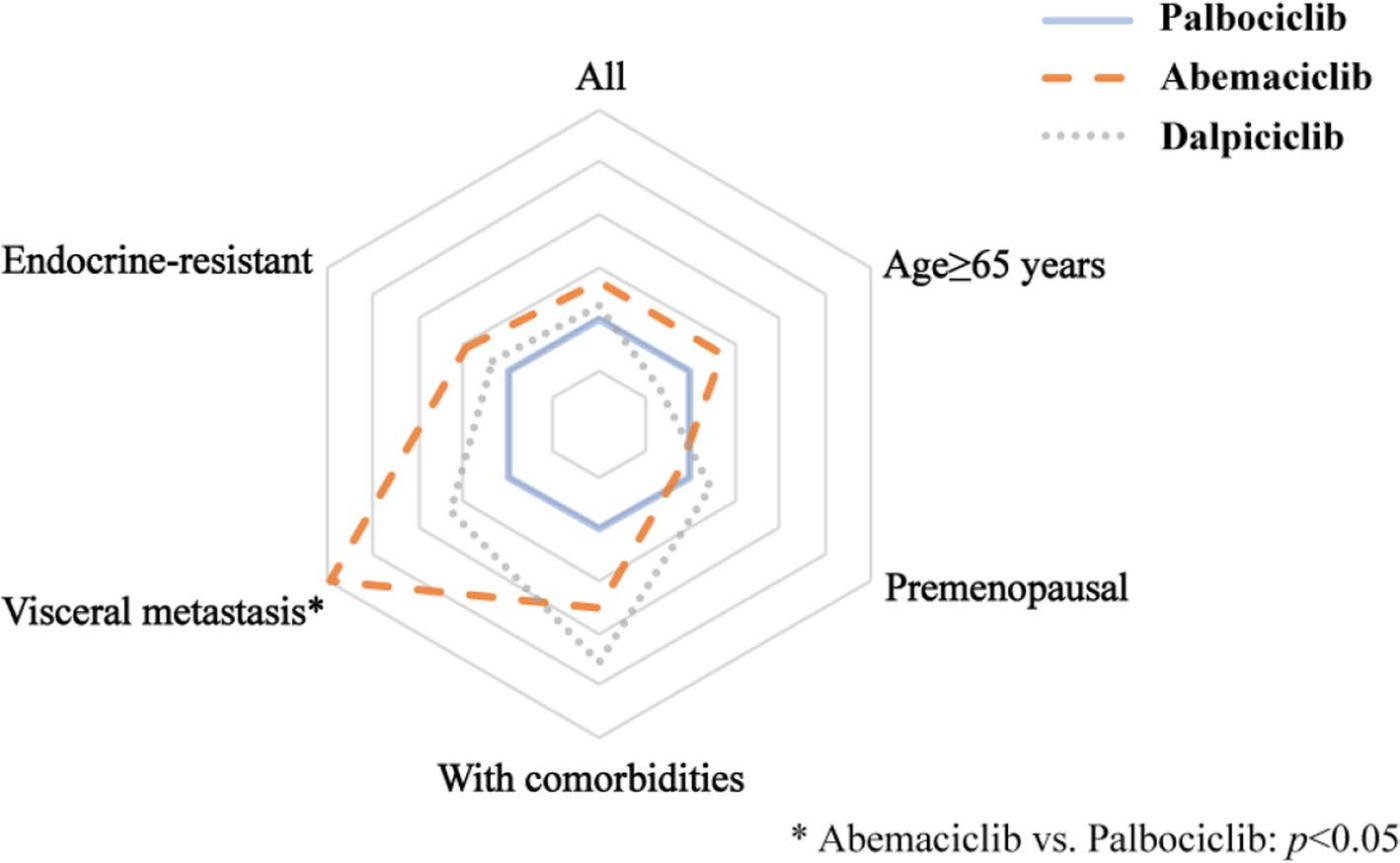
Radar chart of the adjusted odds ratios for tolerability across patient subgroups for three CDK4/6 inhibitors

Among all enrolled patients, dose reduction or discontinuation due to adverse events occurred in 37 cases (20.9%) in the palbociclib group (primarily due to myelosuppression), 34 cases (29.8%) in the abemaciclib group (mainly due to myelosuppression or diarrhea), and 12 cases (24.0%) in the dalpiciclib group (mostly due to myelosuppression). No statistically significant difference was observed in the incidence of dose reduction/discontinuation among the three groups (*p* > 0.05) (Fig. 5). In the subgroup of patients with visceral metastases, the rate of dose reduction/discontinuation was significantly higher with abemaciclib compared to palbociclib (*p* = 0.023). Numerically, patients with underlying conditions such as hypertension, diabetes, or cardiovascular disease exhibited a lower probability of dose modification with palbociclib (palbociclib vs. abemaciclib: adjusted odds ratio = 0.48, 95%CI 0.17–1.39, *p* = 0.178; palbociclib vs. dalpiciclib: adjusted odds ratio = 0.38, 95%CI 0.09–1.56, *p* = 0.180).

## Discussion

According to the latest data from an Italian multicenter real-world study PALMARES-2, abemaciclib demonstrated superior PFS compared to palbociclib as first-line therapy (abemaciclib vs. palbociclib: aHR = 0.71, 95%CI 0.56–0.90, *p* = 0.005). Additionally, the advantage of abemaciclib was also observed in endocrine-resistant patients, luminal B subtype, and premenopausal women (*p* < 0.05), while its efficacy was similar to palbociclib in elderly patients and those with bone-only metastases [10]. These findings are consistent with the results of the present study. Another retrospective study comparing the efficacy of different CDK4/6 inhibitors in first-line treatment also suggested that abemaciclib provided greater PFS benefit than palbociclib in endocrine-resistant patients (NR vs. 17.02 months; *p* < 0.05) [18].

The three CDK4/6 inhibitors share common therapeutic targets but differ in their mechanisms of action, leading to variations in efficacy. CDK4 and CDK6 are key regulators of the cell cycle, activated through binding with D-type cyclins (cyclin D1, D2, and D3) [19, 20]. The activated cyclin D-CDK4/6 complex phosphorylates retinoblastoma protein (RB1), causing dissociation of the RB1-E2F complex and releasing free E2F transcription factors, thereby driving the G1 to S phase transition [21]. Concurrently, free E2F transcription factors promote cyclin E expression, and the resulting cyclin E-CDK2 complex further enhances RB1 phosphorylation, catalyzing G1/S phase progression [22]. As therapeutic agents, CDK4/6 inhibitors target both CDK4 and CDK6 to suppress tumor cell proliferation. Unlike palbociclib, abemaciclib is a multi-target inhibitor capable of inhibiting additional CDKs beyond CDK4/6, including CDK1, CDK2, CDK5, CDK14, and CDK16-18 [23–25]. In preclinical head-to-head comparisons between palbociclib and abemaciclib, abemaciclib demonstrated superior inhibitory activity against CDK4. Given that CDK4 expression is higher than CDK6 in breast cancer tissues and that breast cancer cell growth is more dependent on CDK4 [26], abemaciclib's stronger targeting of CDK4 may contribute to its enhanced efficacy. Moreover, abemaciclib not only blocks the cell cycle but also promotes tumor cell death and regression [27]. In tumor xenograft models, dalpiciclib achieved comparable or even superior antitumor effects to palbociclib by inducing cell cycle arrest and cellular senescence [28]. Consistently, in our previous neoadjuvant study of dalpiciclib, the combination of the dalpiciclib with an aromatase inhibitor demonstrated marked antitumor activity in patients with HER2-negative luminal B breast cancer, achieving a 2-week complete cell cycle arrest (CCCA) rate of 86.7% [29]. These differences in targets and mechanisms among CDK4/6 inhibitors suggest potential variations in their clinical efficacy. Consistent with our findings, abemaciclib outperforms palbociclib in certain patient subgroups with specific clinical characteristics. However, dalpiciclib has not yet shown significant efficacy differences compared to palbociclib, warranting longer follow-up for further evaluation.

Combining the findings from large-scale RCTs (including subgroup analyses in Asian populations) with the results of this study, Asian patients may derive greater PFS benefits from CDK4/6 inhibitors compared to non-Asian patients. A meta-analysis by Lee KWC et al. also suggested that the PFS benefit of CDK4/6 inhibitors combined with ET was more pronounced in Asian populations (hazard ratio = 0.39, 95% CI 0.29–0.51) than in non-Asian populations (HR = 0.62, 95% CI 0.54–0.71) [30]. This discrepancy may be attributed to differences in genetic polymorphisms, epidemiology, and dietary habits across ethnic groups. For instance, Zhou Q et al. identified polymorphisms in the ​​CYP3A4​​ gene among Chinese populations [31]. Reduced CYP3A4 enzymatic activity—critical for hepatic metabolism of CDK4/6 inhibitors [32]—could alter drug exposure. Additionally, Asian breast cancer patients exhibit higher frequencies of somatic mutations in ​​TP53​​ and ​​GATA3​​, as well as ​​ERBB2​​ amplification, compared to non-Asian patients [33]. Beyond cell cycle blockade, CDK4/6 inhibitors enhance T-cell activity and antitumor immunity [34]. Transcriptomic data indicate that immune-related genes (e.g., ​​CD8A​​, ​​PD-L1​​) are more highly expressed in Asian breast tumors than in Western cohorts [33]. Epidemiologically, Asian breast cancer patients are typically younger at diagnosis than their Western counterparts [35], potentially contributing to better drug tolerance. Dietary factors may also play a role: Asians consume more soy-based foods rich in ​​genistein​​, a phytoestrogen that may modulate the activity of palbociclib combined with letrozole [36]. These differences collectively help explain the superior efficacy of CDK4/6 inhibitors in Asian populations. However, even within Asia, genetic and epidemiological profiles vary across countries. Thus, further exploration by Chinese researchers is warranted to clarify the real-world efficacy and population-specific preferences for CDK4/6 inhibitors in China.

Both the adverse event data from this study and published clinical trial results demonstrate that ​​palbociclib​​ and ​​dalpiciclib​​ are associated with a higher incidence of ​​myelosuppression​​, whereas ​​abemaciclib​​ more frequently induces ​​gastrointestinal adverse reactions​​—a distinction attributable to their unique mechanisms of action. Preclinical studies indicate that ​​abemaciclib​​ exhibits relatively weaker inhibition of ​​CDK6​​ compared to ​​CDK4​​ [23, 37, 38], while ​​palbociclib​​ inhibits both kinases comparably [24]. Notably, ​​CDK6​​ (but not CDK4) regulates ​​hematopoietic stem cell differentiation​​ [39, 40], which may explain why palbociclib has a greater propensity for myelosuppression. The gastrointestinal toxicity of abemaciclib likely stems from its off-target inhibition of ​​CDK9​​ and ​​GSK3α/β​​ [41, 42]. These findings suggest that future development of ​​next-generation CDK4/6 inhibitors​​ could mitigate toxicity by minimizing activity against ​​CDK6​​, ​​CDK9​​, and ​​GSK3α/β​​.

Previous retrospective studies on CDK4/6 inhibitors in China have mainly focused on demonstrating the efficacy of single agents, whereas this real-world study compared the efficacy, safety and tolerability of three CDK4/6 inhibitors as first-line treatment for ABC in Chinese patients, while conducting multidimensional subgroup analyses to explore potential differences in efficacy and tolerability among the three drugs under various clinical conditions. However, this study does have several limitations: the small sample size of the dalpiciclib cohort precluded propensity score matching, and coupled with limited follow-up, led to an immature median PFS, warranting cautious interpretation of the observed results; additionally, patients treated with ribociclib were not included into analysis due to insufficient sample size; the varying approval timelines of the three drugs meant patients received treatments during different periods when other available therapeutic options also differed, which may have indirectly contributed to efficacy variations; furthermore, overall survival data were not available due to insufficient follow-up duration, necessitating longer-term follow-up to track these patients' prognostic outcomes. Given the inherent limitations of retrospective studies, future prospective head-to-head comparative studies of different CDK4/6 inhibitors should be conducted in Chinese populations, particularly in clinically relevant subgroups such as endocrine-resistant cases, luminal B subtype, premenopausal patients, and those with visceral metastases where differential efficacy among CDK4/6 inhibitors may be observed, thereby facilitating the development of more personalized treatment strategies tailored for Chinese patients.

## Conclusion

This multicenter real-world study demonstrated that first-line abemaciclib provided superior PFS compared with palbociclib in HR +/HER2 − advanced breast cancer, with overall comparable tolerability across three CDK4/6 inhibitors. The PFS benefit of abemaciclib was particularly evident in premenopausal, PR-positive, HER2-low, luminal B, and endocrine-resistant subgroups. In patients with visceral metastases, abemaciclib offered greater PFS benefit but was associated with poorer tolerability. Safety profiles differed, with higher rates of myelosuppression for palbociclib and dalpiciclib, and more frequent diarrhea for abemaciclib. These findings highlight distinct efficacy–toxicity patterns among CDK4/6 inhibitors, supporting individualized treatment selection in Chinese patients.

## Supplementary Information

Supplementary Material 1.

## Data Availability

Data supporting the findings of this study are available from the corresponding author upon reasonable request.
