# Supplementary material for: Real-world outcomes of CDK4/6 inhibitors in combination with endocrine therapy as first-line treatment for HR-positive/HER2-negative advanced breast cancer: multicenter evidence from China
Source: BMC Cancer. 2026 May 20;26:836. doi: 10.1186/s12885-026-16176-y (PMC13359965; doi:10.1186/s12885-026-16176-y)
Supplement: Supplementary file 1 — Supplementary Material 1. [file 12885_2026_16176_MOESM1_ESM.docx]

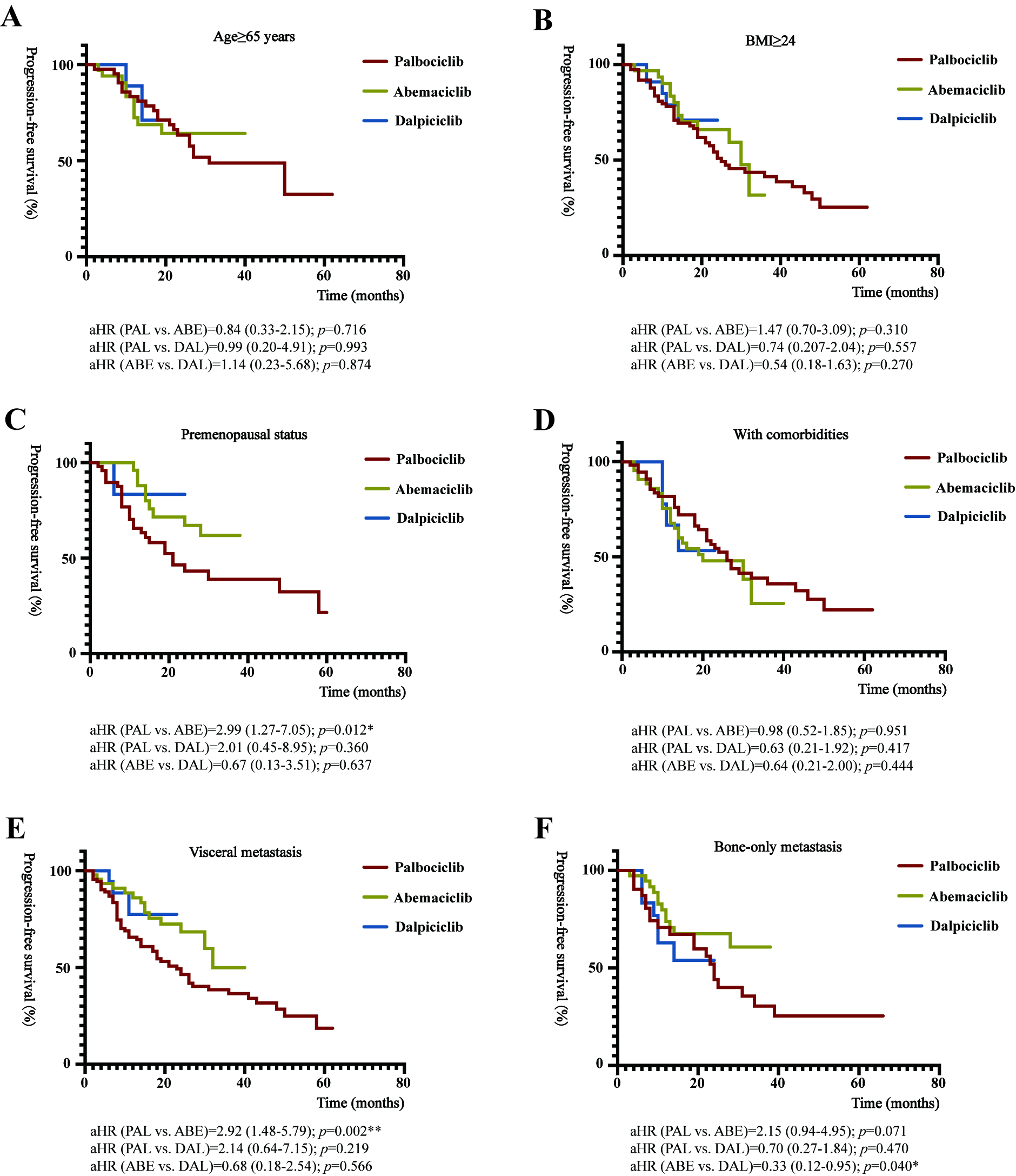


**Supplementary figure 1. Subgroup analysis of progression-free survival for three CDK4/6 inhibitors as first-line therapy in advanced breast cancer​.** (A)​​ Age ≥65 years; (B)​​ BMI ≥24; (C)​​ Premenopausal status; (D)​​ Presence of comorbidities; (E)​​ Visceral metastases; (F)​​ Bone-only metastases.


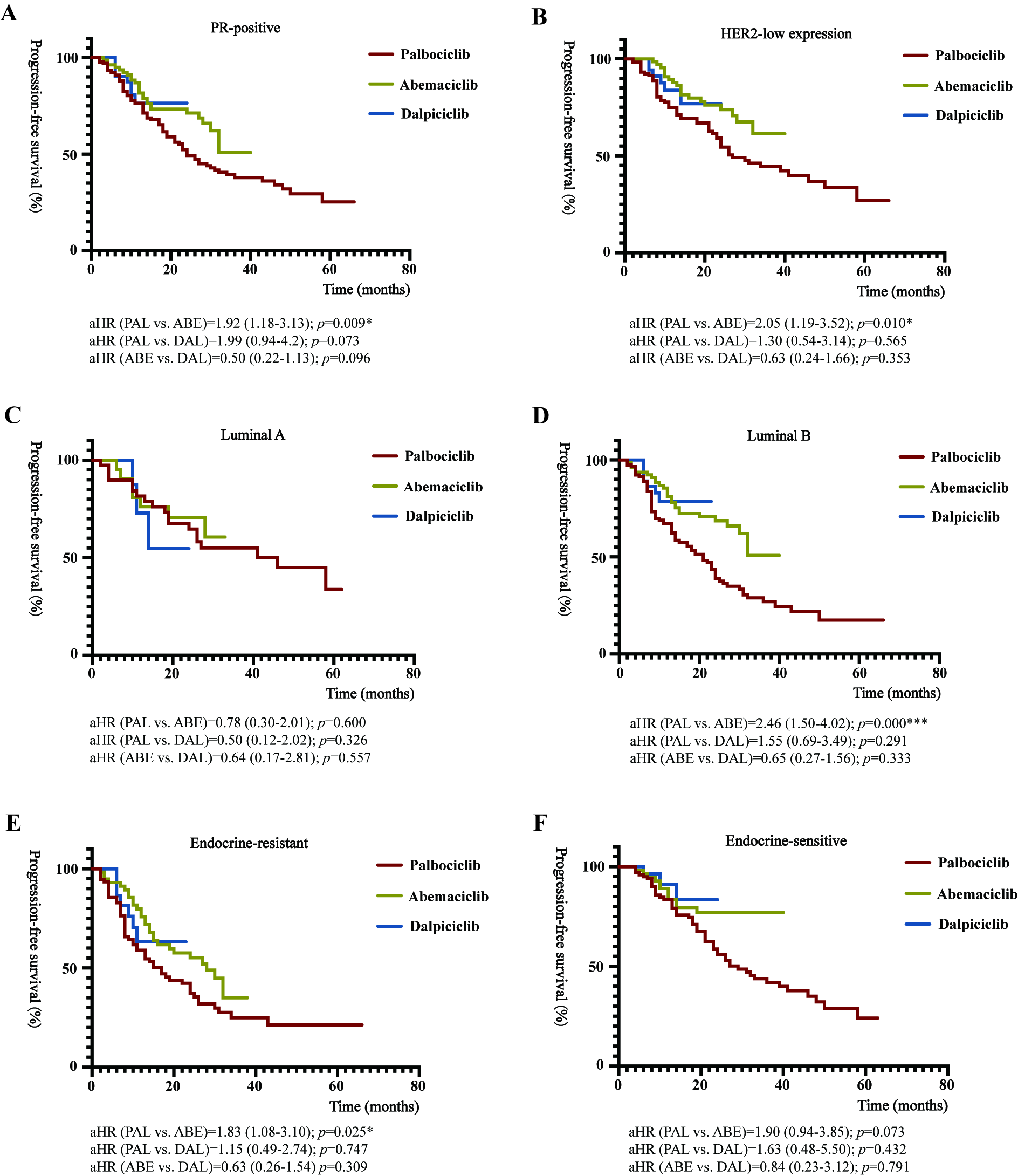


**Supplementary figure 2. Subgroup analysis of progression-free survival for three CDK4/6 inhibitors as first-line therapy in advanced breast cancer​.** (A)​​ ER-positive subgroup; (B)​​ HER2-low subgroup; (C)​​ Luminal A subtype; (D)​​ Luminal B subtype; (E)​​ Endocrine-resistant disease; (F)​​ Endocrine-sensitive disease.


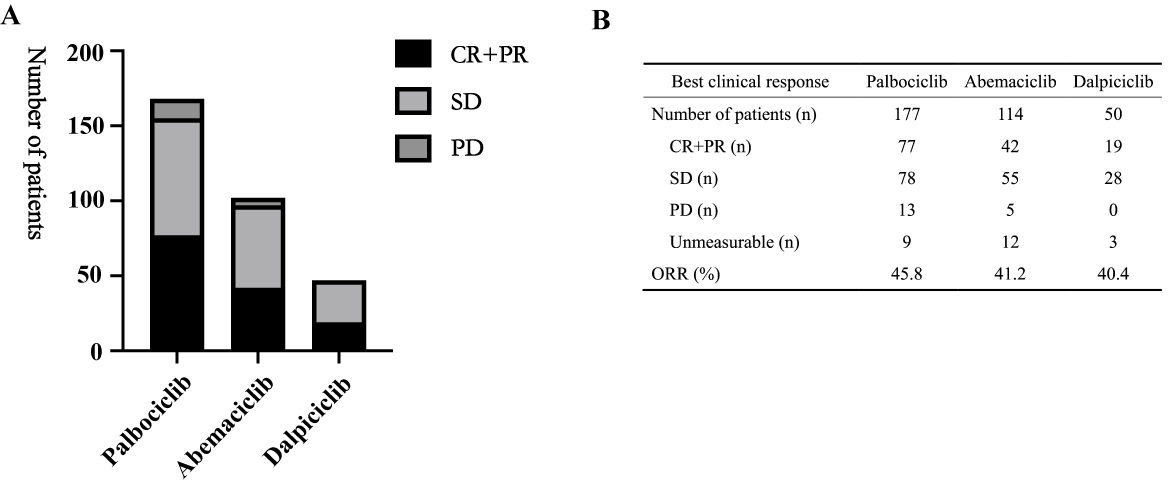


**Supplementary figure 3. Clinical response evaluation of three CDK4/6 inhibitors​.**

Supplementary table 1. Univariate and multivariate analysis of factors influencing progression-free survival with CDK4/6 inhibitors as first-line therapy in advanced breast cancer​

| Factors | Univariate analysis | | |  | Multivariate analysis | | |
| --- | --- | --- | --- | --- | --- | --- | --- |
|  | Hazard ratio | 95%CI | *p* value |  | Hazard ratio | 95%CI | *p* value |
| CDK4/6 inhibitor |  |  |  |  |  |  |  |
| Palbociclib | 1.000 |  |  |  | 1.000 |  |  |
| Abemaciclib | 0.620 | 0.429-0.896 | 0.011* |  | 0.517 | 0.342-0.780 | 0.002** |
| Dalpiciclib | 0.606 | 0.313-1.173 | 0.137 |  | 0.731 | 0.364-1.467 | 0.378 |
| Age |  |  |  |  |  |  |  |
| < 65 years | 1.000 |  |  |  | 1.000 |  |  |
| ≥ 65 years | 0.677 | 0.461-0.995 | 0.047* |  | 0.672 | 0.435-1.037 | 0.073 |
| Menopausal status |  |  |  |  |  |  |  |
| Premenopausal | 1.000 |  |  |  | - | - | - |
| Postmenopausal | 1.091 | 0.757-1.574 | 0.641 |  | - | - | - |
| Male | 0.895 | 0.123-6.538 | 0.913 |  | - | - | - |
| ECOG PS score |  |  |  |  |  |  |  |
| 0-1 | 1.000 |  |  |  | - | - | - |
| ≥2 | 1.023 | 0.600-1.743 | 0.943 |  | - | - | - |
| With comorbidities |  |  |  |  |  |  |  |
| No | 1.000 |  |  |  | - | - | - |
| Yes | 1.272 | 0.922-1.755 | 0.143 |  | - | - | - |
| Disease status |  |  |  |  |  |  |  |
| Stage IV at diagnosis | 1.000 |  |  |  | 1.000 |  |  |
| DFS<24months | 2.203 | 1.276-3.802 | 0.005** |  | 1.161 | 0.569-2.371 | 0.682 |
| DFS≥24months | 1.106 | 0.697-1.755 | 0.668 |  | 0.728 | 0.423-1.252 | 0.251 |
| ER expression |  |  |  |  |  |  |  |
| <50% | 1.000 |  |  |  | 1.000 |  |  |
| ≥50% | 0.471 | 0.286-0.775 | 0.003** |  | 0.621 | 0.361-1.067 | 0.084 |
| PR expression |  |  |  |  |  |  |  |
| negative | 1.000 |  |  |  | 1.000 |  |  |
| positive | 0.691 | 0.482-0.990 | 0.044* |  | 0.789 | 0.533-1.166 | 0.235 |
| Molecular subtype |  |  |  |  |  |  |  |
| Luminal A | 1.000 |  |  |  | - | - | - |
| Luminal B | 1.498 | 0.991-2.266 | 0.055 |  | - | - | - |
| Liver metastasis |  |  |  |  |  |  |  |
| No | 1.000 |  |  |  | 1.000 |  |  |
| Yes | 1.866 | 1.258-2.769 | 0.002** |  | 1.776 | 1.157-2.727 | 0.009** |
| Lung metastasis |  |  |  |  |  |  |  |
| No | 1.000 |  |  |  | - | - | - |
| Yes | 0.867 | 0.616-1.221 | 0.415 |  | - | - | - |
| Bone-only metastasis |  |  |  |  |  |  |  |
| No | 1.000 |  |  |  | - | - | - |
| Yes | 1.041 | 0.724-1.496 | 0.829 |  | - | - | - |
| Visceral metastasis |  |  |  |  |  |  |  |
| No | 1.000 |  |  |  | - | - | - |
| Yes | 1.087 | 0.793-1.489 | 0.605 |  | - | - | - |
| Endocrine resistance |  |  |  |  |  |  |  |
| No | 1.000 |  |  |  | 1.000 |  |  |
| Yes | 1.859 | 1.354-2.552 | 0.000*** |  | 1.503 | 0.956-2.362 | 0.078 |
| Endocrine backbone |  |  |  |  |  |  |  |
| Aromatase inhibitor | 1.000 |  |  |  | 1.000 |  |  |
| Fulvestrant | 1.780 | 1.296-2.446 | 0.000*** |  | 1.443 | 0.961-2.167 | 0.077 |
